# Erosive esophageal reflux vs. non erosive esophageal reflux: oral findings in 71 patients

**DOI:** 10.1186/s12903-015-0069-8

**Published:** 2015-07-25

**Authors:** Herbert Deppe, Thomas Mücke, Stefan Wagenpfeil, Marco Kesting, Anna Rozej, Monther Bajbouj, Anton Sculean

**Affiliations:** Department of Oral and Maxillofacial Surgery, Technical University of Munich, Klinikum rechts der Isar, Ismaninger Strasse 22, 81675 München, Germany; Institute for Medical Biometry, Epidemiology and Medical Informatics, University of Saarland, Homburg/Saar, Germany; Department of Internal Medical Department II, Technical University of Munich, Klinikum rechts der Isar, München, Germany; Department of Periodontology, University of Berne, Bern, Switzerland; Klinik und Poliklinik für Mund-Kiefer-Gesichtschirurgie, Technische Universität München, Klinikum rechts der Isar, Ismaninger Strasse 22, D-81675 München, Germany

**Keywords:** GERD, Oral mucosa, Periodontitis

## Abstract

**Background:**

The purpose of this clinical study was to assess the prevalence of acidic oral mucosal lesions and periodontal conditions in patients suffering from erosive esophageal reflux disease (ERD) compared with non erosive esophageal reflux disease (NERD) patients, both treated with long term proton pump inhibitors (PPI).

**Methods:**

Seventy-one patients with diagnosed GERD were studied: i.e. 29 ERD and 42 NERD patients. Thorough visual examination of the oral mucosa and a periodontal evaluation was performed. The primary outcome was defined as a statistically significant difference, between the two groups, in the presence of acidic lesions of the oral mucosa and specific periodontal parameters.

**Results:**

This study failed to demonstrate statistically significant differences between ERD and NERD patients with respect to the prevalence of oral mucosal lesions. However, significantly more ERD patients suffered from severe periodontitis (CAL ≥ 5 mm) as compared to NERD patients. Accordingly, it may be assumed that PPI-use had no adverse effects on the prevalence of acidic oral mucosal lesions and on periodontal destruction.

**Conclusions:**

Within the limitations of this study it may be concluded that ERD and NERD patients need separate evaluation with respect to periodontal destruction. Moreover, long term PPI medication had no adverse clinical impact on acidic oral mucosal lesions and periodontal destruction. Further studies are necessary to elucidate the role of reflux in the periodontal destruction of ERD individuals.

## Background

One of the most important clinical conditions for retrograde movement of gastric acid into the oesophagus is the gastroesophageal reflux disease (GERD) affecting approximately 10–20 % of the population in the western world [1]. Clinically, typical esophageal symptoms of GERD can occur such as heartburn and acid regurgitation, while on the other hand atypical symptoms such as a burning feeling on the tongue and oral mucosa can be found [2].

However, GERD patients are not a homogenous group. According to the endoscopic diagnosis, an erosive esophagitis (ERD) and a non erosive reflux disease (NERD) may be differentiated. These two main phenotypes of GERD appear to have different pathophysiological and clinical characteristics [3]. The standard therapeutic medical therapy of both phenotypes of GERD includes the administration of acid-suppressive agents, proton pump inhibitors (PPI) [4]. However, erosive esophagitis and NERD clearly diverge when it comes to response to antireflux treatment. NERD patients have a significantly lower response rate to proton pump inhibitor (PPI) therapy, and consequently they constitute the majority of the refractory heartburn group [3].

Recent literature has pointed out that other extraesophageal symptoms of GERD are acidic lesions of the oral mucosa. It has been demonstrated histologically in rats [5] that gastric acid reflux can cause acidic lesions of the palatal mucosa. These findings suggested that these pathological changes may reflect the relationship between laryngopharyngeal reflux and airway obstruction also in humans. Moreover, GERD was reported to be associated with microscopic alterations in the palatal mucosa, such as epithelial atrophy and increased fibroblast numbers [6]. In addition, objective oral mucosal changes were found to be significantly associated with GERD [7]. Also Järvinen et al. pointed out the presence of burning mouth, aphthoid lesions and hoarseness in patients with disorders of the upper digestive tract. Erythema of the soft palate and uvula, glossitis, epithelial atrophy, xerostomia could be common in GERD patients [8]. However, it was objected that the mucosal changes described are quite common and not pathognomonic and specific of GERD patients [9, 10]. Similarly, in a clinical study on 117 patients with reflux disease, no mucosal changes could be observed to be linked with the reflux disease [11].

Accordingly, it may be assumed that these controversial findings are attributable to different proportions of ERD and NERD patients in the respective studies. Nevertheless, in most studies on oral findings, GERD patients were not subdivided in the two subgroups. Similarly, most recent literature has stated that GERD was independently associated with an increased incidence of chronic periodontitis; however, the two phenotypes of GERD were not evaluated separately [12]. Therefore, the purpose of this study was to determine if ERD patients show different oral soft tissue findings and periodontal conditions as compared to NERD patients, both with ongoing PPI therapy.

## Material and methods

### Ethic statement

All clinical investigations and procedures have been conducted according to the principles expressed in the Declaration of Helsinki. Patient informed consent was written. The study was approved by the local ethic committee of the Klinikum rechts der Isar.

### Patients

**Fig. 1:**
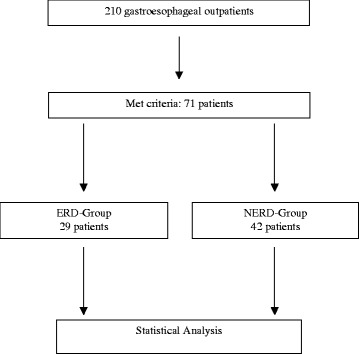
Flow chart of patients

From March 2009 to March 2010, a total of 201 gastroesophageal outpatients of the Department of Internal Medicine II (Head: Univ.-Prof. Dr. R. M. Schmid) of this University who were at least 18 years of age were informed by the internist on the purposes of this study. Inclusion criteria were patients who had shown evidence for GERD on functional testing (pH monitoring combined with impedance measurement) and/or the confirmation of an erosive form (esophagogastroduodenoscopy). Moreover, included patients had to have at least two molars, two premolars and four anterior teeth free of prosthetic crown restoration in each jaw. Patients were excluded from the study if they had removable dentures, had undergone radiotherapy in the head and neck area, bisphosphonate therapy, or had a history of alcohol or illicit drug use. In consequence, a total of 71 patients met criteria and provided informed consent for oral evaluation (Fig. 1). During evaluation, ethnicity, number of teeth and smoking habits (yes/no) were recorded. To eliminate bias, the only dentist (A. R.) who performed all the dental exams was blinded. Hence, she did not know whether a particular patient had been diagnosed with ERD or NERD.

### Medical history

All patients had to show a history of long term PPI therapy (>1 year). In addition, details of the health status were assessed.

### Oral mucosa

Acidic mucosal lesions were examined in the palatal, buccal, and tongue mucosa using the following scoring system: score 0, no inflammation; 1, redness; and 2, ulceration [13]. Accordingly, presence of erythema and/or ulcer was included in the definition of acidic mucosal lesions. Inspection was performed with use of a loup (Carl Zeiss, Jena, Germany, magnification × 2.3).

### Periodontal parameters

Oral hygiene was measured for each tooth according to the criteria for the Plaque Index [14]. In this Index, a score of 0 means no plaque and score 1 that a film of plaque is adhering to the free gingival margin and adjacent area of the tooth. Moderate accumulation of soft deposits within the gingival pocket, or the tooth and gingival margin which can be seen with the naked eye, is assigned to score 3. Abundance of soft matter within the gingival pocket and/or on the tooth and gingival margin is assigned to score 4. For assessment of bleeding sites which indicate local inflammation, a modified bleeding on probing index (BOP) was scored as percentage of sites that showed bleeding 30 seconds after gentle probing of the bottom of the pockets [15]. The clinical attachment loss (CAL) was measured with the WHO probe (Morita, Kyoto/Japan) [16] from the cemento- enamel junction to the bottom of the pocket on four sites (mesio-vestibular, disto-vestibular, mesio-oral and disto-oral) [17]. Severity of periodontal condition was described for all the individual sites as a whole and was categorized on the basis of clinical attachment loss as severe in case of CAL ≥ 5 mm [18].

### Documentation

All parameters and findings were documented in standardized forms, which allowed comparison. Oral mucosa was documented by digital photographs (Finepix AX 300, FUJIFILM Europe GmbH, D-Düsseldorf).

### Statistical analysis

Statistical analysis was performed using a commercial computer program (NeoOffice 3.1.1, Planamesa Inc., 123 Main St. Sunnyvale, California, USA and StatXact-program, version 5.0.3, Cytel Inc., Cambridge, Massachusetts, USA). Statistical significance was tested by the Chi-square test and Student’s t test. A p-value less than 0.05 was considered to indicate statistical significance.

## Results

### Patients

The study encompassed a total of 71 patients, 41 women and 30 men, with a mean age of 49.7 years (SD ± 15.1 years, range: 22–83 years). In detail, the ERD group consisted of 29 patients and comprised 16 women and 13 men with a mean age of 48 ± 16 years (range: 22–83 years). In the NERD group, 25 women and 17 men were included with a mean age of 50.9 ± 14.5 years (range: 25 – 74 years).

With respect to conservative dental restorations (fillings, endodontic treatment) or fixed prosthetic dental reconstructions, there was no significant difference between the ERD and NERD patients. Study participants in both groups consisted in all cases of Caucasian individuals. With respect to the number of teeth and smoking habits, there were no significant differences (Table 1). All smokers in this study used to smoke cigarettes. No other forms of tobacco such as spit tobacco, cigars and pipes were consumed.

**Table 1:** Initial data of the ERD and NERD group. No statistical significance between the two groups detectable

Parameter	ERD group (*n* = 29)	NERD group (*n* = 42)	*p*	Difference significant?
Age (mean ± SD)	48.0 ± 16.03	50.9 ± 14.5	0.43	No
Range (years)	22–83	25 – 74		
Female (*n* = 41)	16	25	0.22	No
Male (*n* = 30)	13	17	0.22	No
Caucasian ethnicity	100 % (*n* = 29)	100 % (*n* = 42)	0	No
Teeth (mean ± SD)	20.6 ± 8.4	18.8 ± 7.6	0.35	No
Smoking	7 (24.1 %)	12 (28.6 %)	0.17	No

### Medical history

With respect to medical care, all study patricipants had provided name and address of their family doctors and family dentists and had confirmed to see them regularely. According to the inclusion criteria, all 71 patients (100 %) were prescribed periodically PPI over the long term (>1 year, mean: 1.3 years ± 4.5 months; range: 1.0–3.2 years) which was ongoing at the time of evaluation. However, three of them in the NERD group were classified as non-responders. In addition, medical history of the 71 patients revealed no impact on oral mucosa.

### Oral mucosa

A total of eight patients in the ERD group and 11 NERD patients showed moderate erythema of the oral mucosa (score 1). In both groups, erythemas were localized equally on the palatal and buccal mucosa and the mucosa of the tongue. No patient demonstrated ulcer of the mucosa. Statistical analysis failed to demonstrate significant differences between scores and groups (Table 2).

**Table 2:** Acidic oral mucosal lesions palatal (PM), buccal (BM), and tongue mucosa (TM). No statistical significance between scores and groups

Clinical symptom	ERD group (*n* = 29)	NERD group (*n* = 42)	*p*	Difference significant?
PM score 0	25 (86.2 %)	36 (85.7 %)	0.95	No
PM score 1	4 (13.8 %)	6 (14.3 %)	0.95	No
PM score 2	0	0	1	No
BM score 0	27 (93.1 %)	38 (90.5 %)	0.69	No
BM score 1	2 (6.9 %)	4 (9.5 %)	0.69	No
BM score 2	0	0	1	No
TM score 0	27 (93.1 %)	40 (95.2 %)	0.70	No
TM score 1	2 (6.9 %)	2 (4.8 %)	0.70	No
TM Score 2	0	0	1	No

### Periodontal parameters

With regard to the oral plaque index, similar levels of oral hygiene were found in both groups. ERD patients showed a higher percentage of BOP as compared to NERD patients. However, statistical analysis failed to demonstrate significance on the 5 % level. Similarly, mean CAL values showed no significant differences. However, significantly more ERD patients suffered from severe periodontitis (CAL ≥ 5 mm) as compared to NERD patients (Table 3).

**Table 3:** Periodontal findings. Significantly more patients suffering from severe periodontitis in the ERD group

Parameter	ERD group (*n* = 29)	NERD group (*n* = 42)	*p*	Difference significant?
Plaque index	0.76 ± 0.74	0.5 ± 0.55	0.09	No
(Mean ± SD)				
BOP (%)	35.5 ± 16.4	31.3 ± 18.2	0.32	No
(Mean ± SD)				
CAL (mm)	7.4 ± 1.6	6.5 ± 0.5	0.35	No
Severe periodontitis	73.1 % (22/29)	35.7 % (15/42)	*p* < 0.001	Yes
(CAL ≥ 5 mm)				

## Discussion

Recent literature has pointed out that, with respect to GERD patients, only controversial epidemiological data on the prevalence of acidic oral mucosa lesions are available [9]. Moreover, most recent literature demonstrated that GERD is independently associated with an increased incidence of chronic periodontits. However, GERD and periodontitis are frequent chronic diseases and PPI medication generates costs for the public health care system. Therefore, clinical studies are urgently necessary to find out if there is an association between the main two subgroups of GERD and acidic oral mucosal lesions (erythema or ulcer) and periodontal conditions with respect to PPI medication.

Several studies on oral findings in GERD patients provide only limited information if or how GERD was diagnosed (endoscopy solely [19], esophageal pH monitoring in combination with impedance measurement [20]) or treated before the study was carried out [19, 21–23], which hampers reliable comparison with the present results. In this study, all patients had shown evidence for GERD on functional testing (pH monitoring combined with impedance measurement) and/or the histological confirmation of an erosive GERD (esophagogastroduodenoscopy). Moreover, in this study, both groups of GERD patients were treated with PPI for at least 1 year which allows conclusions on the clinical impact on oral conditions of PPI in this sample of patients.

With respect to the dental status, patients with removable dentures were not excluded in all studies, which, again, hampers comparison of the present results with the literature. In a recent study [13], three patients with GERD were completely edentulous; however, one participant, a 72-year old woman in the GERD group, was found to have a mucosal lesion, a small ulcer-like lesion associated with redness on the dorsal tongue. Accordingly, it remains unclear if ulcer was attributable to the prosthodontic construction or to GERD. In this study, none of the subjects had removable dentures to eliminate bias if mucosal alterations were caused by the prosthodontic construction.

Although GERD affects all age groups [21], the incidence of this disease increases considerably after 40 years of age [19]. Similarly, in this study, the mean age of patients was 48.0 ± 16.03 years in the ERD group vs. 50.9 ± 14.5 years in the NERD patients, which is in the order published in the literature [20]. Moreover, with respect to age, there was no statistically significant difference between the ERD and the NERD group in this study. In accordance with the literature, among all 71 GERD patients, a total of 41 (55.7 %) were females. Also in previously published studies, a higher incidence of GERD in women has been reported [23]. With respect to ethnicity, number of teeth and smoking habits, no differences were found. Due to the fact that the smokers among the study participants did not consume other forms of tobacco such as spit tobacco, cigars or pipes, the study focussed on “cigarette smoking”. Therefore, due to the fact that the aforementioned characteristics were very similar in both groups, the results of this study seem not to be compromised by these general factors.

Due to the fact that all study participants had confirmed to attend regularely medical and dental care, it seems unlikely that periodontal findings in this study may be attributed to lack of accessing routine dental care including dental prophylaxis. All 71 patients (100 %) were prescribed periodically PPI over the long term. However, three patients of the NERD group were classified as non responders. Clinical efficacy of PPI medication has been documented by Wang and coworkers who collected gastric fluid during routine endoscopy in patients on PPIs, on H2-receptor blockers and on no acid suppression therapy [24]. The mean pH values were 5.11, 4.12 and 2.91, respectively. However, a recent study has PPI even proved to be ineffective in a number of patients [2]. Data from a meta analysis have shown that a high-dose proton pump inhibitor is no more effective than placebo in producing symptomatic improvement or resolution of laryngo-pharyngeal symptoms [25]. Accordingly, the three non responders found in this study are in accordance with the literature.

Many investigators have proposed an association between GERD and laryngo-pharyngeal symptoms such as hoarseness, globus pharyngeus, vocal fatigue, frequent sore throat, frequent throat clearing, chronic cough [26–30]. Moreover, oral mucosal lesions may result from GERD by direct acid or acidic vapor contact in the oral cavity [9].

It has been demonstrated histopathologically in the rat model that reflux affects the soft palate, which suggests that these pathological changes may reflect the relationship between laryngopharyngeal reflux and airway obstruction [5]. One clinical large case-controlled study observed a significant association of GERD with erythema of the palatal mucosa and uvula [7]. In another study, histologic examination of palatal mucosa found a greater prevalence of epithelial atrophy, deepening of epithelial crests in connective tissue and a higher prevalence of fibroblasts in 31 GERD patients compared with 14 control subjects [6]. But, these changes were not visible to the naked eye, unlike the mucosal changes that may be more readily observed in esophagitis and laryngitis where the pH of the gastric reflux at these sites is lower than in the mouth [31, 32]. Other studies have not found any abnormal appearances of the oral mucosa or associated oral symptoms in patients with confirmed GERD [8, 11].

Also in this study, there was no statistical significant difference with regard to the total number of oral mucosal lesions and their localization when the ERD and the NERD group were compared. However, there is a paucity of information on the effect of GERD and PPI on oral mucosal changes in the literature. Acid regurgitation may exacerbate oral mucosal changes associated with co-existing hyposalivation, which can arise from systemic conditions, local salivary gland conditions and intake of drugs including PPIs [9]. PPIs inhibit the H^+^/K^+^-ATPase pump in the stomach and other tissue [33]. Altman and coworkers have demonstrated the presence of this pump in laryngeal seromucinous glands [34]. In addition, there is evidence that systemic medication may enter saliva through diffusion [35]. Thus, it is possible for the pH of the seromucinous secretions to be affected by PPI use, and this could alter the oral mucosa, and, in addition, the bacteria growth environment in the oropharynx [33]. Especially, patients with diabetes and a history of recent PPI use are more likely to have abnormal oral flora [33]. However, due to the fact that lesions of the oral mucosa did not differ significantly between the ERD and the NERD group in the current study it may be assumed that PPI medication had no adverse impact on oral mucosal health in both groups.

Periodontal evaluation consisted of clinical three dimensional evaluation by probing due to the fact that two dimensional radiographs are not highly reflective of the real periodontal situation [36]. With respect to the oral plaque index, bleeding index and clinical attachment loss, similar levels were found in both groups (Table 3). Differences between these two groups were not statistically significant on the 5 % level. However, significantly more ERD patients suffered from severe periodontitis (CAL ≥ 5 mm) as compared to NERD patients. This is, in part, in accordance with the most recent literature. Also Song and coworkers [12] have shown that GERD was independently associated with an increased incidence of chronic periodontitis. The most reasonable explanation for GERD as a predisposing factor for chronic periodontitis would be poor salivary function [12], which has been demonstrated in GERD patients in several papers [7, 37, 38]. Nevertheless, hyposalivation can explain the present findings only in part, because incidence of severe periodontitis was different in ERD and NERD patients. Accordingly, it may be assumed in concordance with the mucosal findings in this study that PPI medication had no adverse effect on periodontal health in ERD and NERD patients. Other parameters such as more aggressive acidic reflux must contribute to the more severe periodontal destruction in ERD patients. Unfortunately, to our knowledge, there is no similar study in the literature available on periodontitis in ERD and NERD patients. Therefore, further studies are available in GERD subgroup patients.

This study has some limitations. First, patients were recruited from an outpatient setting of a university hospital. Therefore, it cannot be excluded that patients are not representative for the whole population. Second, salivary gland function has not been evaluated. It can not be excluded completely that ERD and NERD patients show different degrees of hyposalivation. Third, frequency and chronicity of use of tobacco were not recorded. Accordingly, it can not be excluded that frequency and chronicity of use of tobacco differed significantly between the two groups (ERD, NERD). Four, erythema and ulceration of the oral mucosa is a very common finding with multiple confounding etiologies. It can not be excluded that some lesions in this group of GERD patients are caused by other origin and not by reflux acid.

## Conclusions

Within the limitations of this study, the authors failed to demonstrate significant differences between ERD and NERD patients with respect to the prevalence of acidic lesions of the oral mucosa and their localization. Therefore, the association of GERD and oral erythema could not be demonstrated in the present patients. In contrast, the results indicated that ERD patients may suffer from more severe periodontitis as compared to NERD patients. However, the study failed to show a clinical impact of long term PPI medication on the prevalence of acidic oral mucosal lesions and periodontal destruction. Further studies are necessary to elucidate the role of reflux in the periodontal destruction of especially ERD individuals.

## Abbreviations

BOP: Bleeding on probing index
CAL: Clinical attachment loss
ERD: Erosive esophageal reflux disease
NERD: Non erosive esophageal reflux disease
PI: Plaque index
PPI: Proton pump inhibitors
WHO: World Health Organisation
